# The Study of Cost-Effectiveness of Rivaroxaban versus Warfarin in Patients with Atrial Fibrillation Who Developed Ischemic Stroke

**DOI:** 10.1155/2021/5534873

**Published:** 2021-09-07

**Authors:** Neda Jaberi, Zahra Kavosi, Etrat Hooshmandi, Nasrin Moradi, Khosro Keshavarz, Afshin Borhani-Haghighi

**Affiliations:** ^1^Health Human Resources Research Center, School of Health Management and Information Sciences, Shiraz University of Medical Sciences, Shiraz, Iran; ^2^Clinical Neurology Research Center, Shiraz University of Medical Sciences, Shiraz, Iran

## Abstract

**Introduction:**

Rivaroxaban is a new anticoagulant providing benefits for the treatment of patients with atrial fibrillation (AF). This study is aimed at evaluating the cost-effectiveness of rivaroxaban compared to warfarin in patients with AF.

**Method:**

This economic evaluation study was conducted among 144 selected nonrandomly patients who were treated with rivaroxaban or warfarin and suffered from AF leading to stroke, in the stroke ward of Shiraz Nemazee Hospital in 2019. The final and clinical (intermediate) outcomes were QALYs and no bleeding and prevention of ischemic stroke, respectively. The study was performed from the social perspective, and a deterministic one-way sensitivity analysis was conducted to identify the effects of uncertainty. The analysis of the collected data was carried out using SPSS18 and TreeAge software.

**Results:**

Patients who received rivaroxaban had lower costs ($ 25275 vs. $ 26554) and higher QALYs (0.5 vs. 0.33) compared to those taking warfarin. Bleeding and stroke occurred in (9 vs. 40) and (1 vs. 3) patients in the rivaroxaban and warfarin groups, respectively, and there was a significant decrease in the incidence of bleeding in the rivaroxaban group (81.9% vs 44.4%). Thus, rivaroxaban in all the outcomes was cheaper and more effective than warfarin. The one-way sensitivity analysis confirmed the robustness of the results.

**Conclusions:**

Considering the incremental cost-effectiveness ratio, rivaroxaban is more cost-effective and can be a dominant alternative. Therefore, it is suggested to use rivaroxaban as the first priority in AF patients because rivaroxaban reduces costs and increases clinical outcomes compared with warfarin.

## 1. Introduction

Stroke is among the most common causes of mortality and morbidity particularly in developing countries [1]. Stroke mortality and morbidity were much higher in low-income and middle-income countries (LMICs) than high-income countries (HICs) [2]. At least, one of four patients with ischemic stroke had cardioembolic source in our previous series [3]. Atrial fibrillation (AF) is one of the most prevalent sources of cardiac embolism [4]. Patients suffering from thromboembolic stroke have a higher fatality, morbidity, and longer hospitalization due to AF compared to stroke patients due to another causes [5].

Warfarin has been traditionally used for prevention of recurrent stroke in patients with AF. However, warfarin has a narrow therapeutic index, high risk of bleeding, and drug-drug and dietary interactions. Moreover, heparin or heparinoids should be administered before warfarin as bridging therapy in some instances [6]. Compared to warfarin, direct acting oral anticoagulants (DOACs) such as dabigatran, rivaroxaban, apixaban, and some other drugs have not had abovementioned limitations of warfarin. Howbeit, their direct cost has been significantly higher [7].

Cost-effectiveness analysis is a method of economic evaluation in which the value of the resources spent on an intervention is compared with the extent of health obtained from that intervention. Cost-utility analysis is a form of cost-effectiveness analysis, in which the utility is used instead of natural units to measure outcomes [8]. Although there have been several studies regarding with cost-effectiveness of rivaroxaban compared in comparison to warfarin [9–13], there is spare data from LMICs, and accordingly, we investigated direct medical costs, direct nonmedical costs, and indirect costs of patients who had ischemic stroke and AF and taking warfarin or rivaroxaban to compare cost-effectiveness of these drugs.

## 2. Patients and Methods

### 2.1. Study Design and Population

This cross-sectional, prospective, and nonrandomized observational trial study is an economic evaluation conducted in September 2018 to September 2019 at Nemazee Hospital in Shiraz. This is a high-volume referral center in South of Iran. In this study, 144 consecutive patients were enrolled and followed for one year.

Stroke is defined as an episode of focal neurologic deficit with acute onset and lasting more than 24 hours (or lasting <24 hours with imaging evidence of ischemic infarct corresponding with current symptoms). Brain CT and/or MRI was performed on all patients to define the infarction territory and exclude cerebral hemorrhage. The inclusion criteria for the study were (1) patients with a recent diagnosis of first-ever ischemic stroke [14] and (2) patients aged between 15 and 50 years. We excluded patients who suffered from intracerebral hemorrhage, head trauma and subarachnoid hemorrhage, patients with transient ischemic attack without radiologic confirmation, large arteria diseases, lacunar stroke, vasculitis, arterial dissection, fibromuscular dysplasia, Moyaoya disease, and sickle cell disease. In addition, patients with mRS > 4; hypersensitivity to warfarin or rivaroxaban; pregnancy; breast feeding; uncontrolled hypertension (blood pressure more than 220/120 mmHg); renal or hepatic failure; and infectious endocarditis, cerebral infarcts due to cardioaortic embolic causes other than AF such as recent myocardial infarction (MI), left ventricular thrombus, valvular heart disease, ejection fraction [15] less than 28%, atrial myxoma, patent foramen ovale, aneurysm of inter atrial septum, mobile thrombus in ascending aorta, or aortic arch were excluded too.

AF confirmed by electrocardiography (ECG) or 24 h Holter monitoring before, during, or after ischemic stroke. Allocation of rivaroxaban or warfarin was done according to patient's preference and under insurance coverage. Rivaroxaban (Xalerban, Abidi company, Tehran, Iran) and warfarin were provided by patients themselves.

Patients were randomly selected from the patients under treatment with anticoagulants referred to the mentioned medical centers. For this purpose, the researcher went to the stroke ward of Nemazee Hospital on a daily basis and evaluated stroke patients due to AF treated with rivaroxaban and warfarin. After reaching the quorum, the sample size for each group was stopped. According to the previous study [16], using the following formula, the sample size was obtained 77 in each group:

(1) $$\begin{array}{c} n_{1}=n_{2}=\frac{\left(p_{1}\left(1-p_{1}\right)+p_{2}\left(1-p_{2}\right)\right){\left(Z_{1-\alpha /2}+Z_{1-\beta }\right)}^{2}}{{\left(p_{1}-p_{2}\right)}^{2}}, \end{array}$$

where *α* = %5, *β* = %20, *Z*1 − *α*2 = 1.96, and *Z*_1−*β*_ = 0.84.

### 2.2. Data Collection

#### 2.2.1. Cost Inputs

The study was performed from the societal perspective. Thus, direct and indirect costs were included. The data of direct medical costs (DMC) were collected through reviewing the patients' medical records and experts' opinions. The direct nonmedical costs (DNMC) and indirect costs (IC) were also collected using the cost collection forms and self-reporting of the patients. The human capital approach was used to calculate indirect costs. The costs were estimated based on tariffs in 2019 for each international dollar (purchasing power parity) with an exchange rate of 128000 Rials per dollar [17].

#### 2.2.2. Clinical Outcomes

Primary clinical outcomes were any type of stroke (ischemic or hemorrhagic); myocardial infarction; intracranial hemorrhage; life threatening nonintracranial hemorrhage which needs blood transfusion; and minor nonintracranial hemorrhage such as gastrointestinal, genitourinary hemorrhage, epistaxis, and ecchymosis or skin hematoma. All patients were informed about the abovementioned adverse effects and requested to call investigators if any adverse event occurred. Routine neurologist follow-up was done every three months. CBC, U/A, stool OB/OP, and renal and liver function tests were done for all patients in three months' interval. For patients who took warfarin, the international normalized ratio (INR) was monitored to evaluate the anticoagulation effect of warfarin. The target INR level was 2.5. Effectiveness was defined as absence of each and any abovementioned adverse outcome.

Also, for calculating the utility values, the EQ-5D generic questionnaire was used which measured the utility scores through telephone calls with 144 patients. EQ-5D has 5 questions and assesses 5 aspects of mobility, self-care, usual activities, pain/discomfort, and anxiety/depression. After completing the EQ-5D questionnaire, we tried to evaluate the national values of Iran which had been directed in a separate study by Goudarzi [18] using time trade-off [19], and we changed the five digit codes of the questionnaire into numerical utility (score 1 indicates the best state of health while zero indicates death). However, since final outcomes are preferred for policy-making and decision-making, only the quality-adjusted life year (QALY) outcome was used for the final analysis, ICER, cost-effectiveness plan, and the one-way sensitivity analysis.

### 2.3. Cost-Effectiveness Analysis

To analyze the collected data, SPSS 18 and TreeAge softwares were used, and then costs, effectiveness, and incremental cost-effectiveness ratio (ICER) were estimated for the two treatment option. The ICER was calculated using the following formula:

(2) $$\begin{array}{c} \text{ICER}=\frac{\text{Cost}\_\text{Rivaroxaban}‐\text{Cost}\_\text{Warfarin}}{\text{Effectiveness}\_\text{Rivaroxaban}‐\text{Effectiveness}\_\text{Warfarin}} \end{array}$$

### 2.4. Uncertainty Analysis

The one-way sensitivity analysis was done to examine the effect of the uncertainty of parameters on the study results. The values of the variables changed by 20%, and the tornado diagrams were drawn. Because of the lack of a specific cost-effectiveness threshold in Iran, the threshold in developing countries for each QALYs, as recommended by WHO, was set at one time and three times of gross domestic product per capita that was about $21011 PPP (GDP × 1) and 63033 (GDP × 3) at 2019 for Iran, according to the World Bank report [20].

### 2.5. Study Ethics

The Ethical Committee of Shiraz University of Medical Sciences approved the study under the approval number of 1397-281. All patients who were included in the trial filled out informed consent forms.

## 3. Results

According to Table 1, there were statistically significant differences between the cost treatment with rivaroxaban and treatment with warfarin. The results showed that although the cost of medication in the rivaroxaban group was higher than the warfarin group (*P* < 0.001), the costs of diagnosis and lab services (*P* < 0.001) and lost income (*P* = 0.005) were lower in the rivaroxaban group. The highest share in both options was related to the mean cost of lost income ($19531 for rivaroxaban and $20864 for warfarin), so that the total costs for rivaroxaban and warfarin were $25275 and $26554, respectively (*P* = 0.004).

Additionally, the mean DMC of visiting a doctor, physiotherapy and other services, and hospitalization in the warfarin group was higher than those of rivaroxaban; however, these differences were not statistically significant. Also, there was no significant difference between the costs of accommodation and travel in the rivaroxaban group compared to the other one.

The results revealed that rivaroxaban obtained better scores for the outcomes studied, including effectiveness (81.9% vs. 44.4% for no bleeding and 98.6% vs. 95/7% for prevention of ischemic stroke) and QALYs (0.5 vs. 0.33), compared to warfarin (*P* < 0.001). 64.9% of patients in the warfarin group and 67.2% in the rivaroxaban group received the medication for one year and the rest for more than one year. 16.7% of patients treated with warfarin had INR higher than normal levels. According to Table 2, base of classification the most common bleeding in patients was minor nonintracranial hemorrhage and among these, the highest percentage was related to ecchymosis in both groups (12.5% in the rivaroxaban group and 36.1% in the warfarin group). Although the incidence of ischemic stroke was higher in the warfarin group than in the other group (%4.3 vs. %1.4), the difference was not significant (*P* > 0.05).

According to Tables 3 and 4, the incremental cost-effectiveness ratio based on the QALYs, prevention of bleeding, and prevention of ischemic stroke was obtained negative for warfarin. Therefore, treatment with rivaroxaban in all the outcomes was the dominant option; it was cheaper and more effective, and for per extra QALY gained, it not only did not impose a higher cost but also saved US $7,523 and $3,365 for no bleeding as well as $42633 for prevention of ischemic stroke status per extra effectiveness unit.

**Figure 1:**
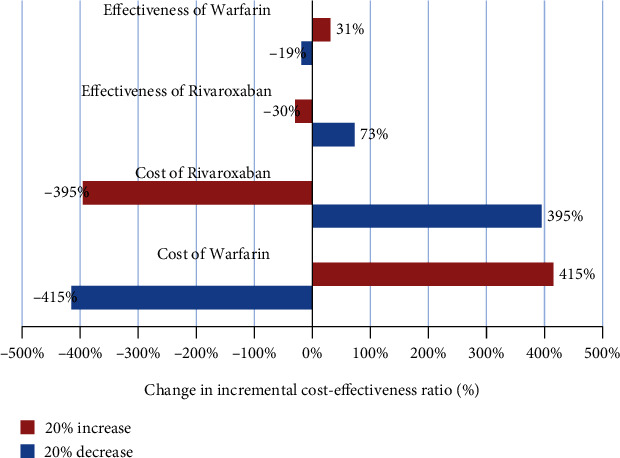
Tornado diagram of cost-effectiveness to prevent of bleeding for patients with AF under treatment with rivaroxaban and warfarin.

**Figure 2:**
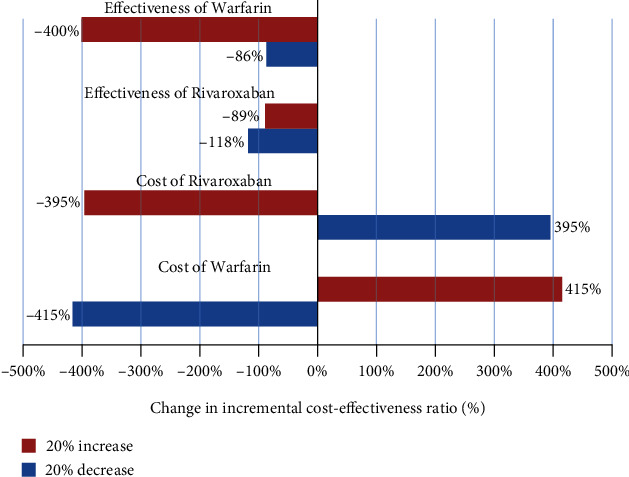
Tornado diagram of cost-effectiveness to prevent of ischemic stroke for patients with AF under treatment with rivaroxaban and warfarin.

**Figure 3:**
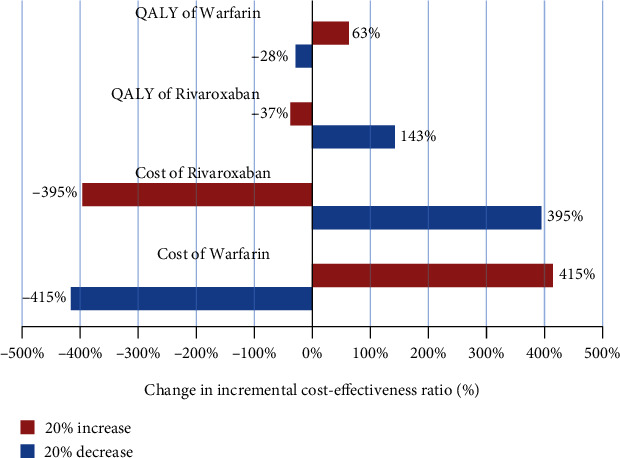
Tornado diagram of cost-utility for patients with AF under treatment with rivaroxaban and warfarin.

The one-way sensitivity analysis in the form of tornado diagrams is illustrated in Figures 1–3. The ICER had the maximum sensitivity to the increased cost of warfarin treatment method in the outcomes of prevention of bleeding, ischemic stroke, and QALYs (Figures 1–3). On the other hand, it had the minimum sensitivity to increased effectiveness and QALYs of rivaroxaban and warfarin. It suggests that rivaroxaban intervention is the dominant option and the most cost effective.

## 4. Discussion

Although there are several studies regarding with cost-effectiveness of NOACs (novel oral anticoagulants) in the prevention of recurrent stroke due to AF in high-income countries [13, 21–23], as far as we know, the current study is one of the few concerning this issue in lower middle-income countries (LMICs) and first comprehensive economic evaluation study on AF patients in Iran and in the EMRO region (Eastern Mediterranean Region). The study is aimed at evaluating new oral anticoagulant strategies for the prevention of ischemic stroke from a societal perspective.

The results of the ICER study based on QALY, no bleeding, and stroke status ($ -7,523, $ -3,365 and $ -42,633) in patients with AF showed that rivaroxaban was the superior and more cost-effective option as it was more effective and less costly than warfarin. This finding is in line with the results of Kleintjens et al. (2013) and Soyon et al. (2012) studies [13, 21].

The results of cost showed that treatment with rivaroxaban and warfarin had a direct, indirect, and total cost of $ 5743 vs. 5689, $ 19531 vs. 20864, and $ 25275 vs. 26554 per a one-year period, respectively. Thus, the cost of a one-year period of treatment per patient treated with rivaroxaban was lower than that of the treatment with warfarin. Further, although the direct costs of NOACs are more than warfarin, due to higher risk of ischemic and hemorrhagic complications and given that most of them were self-employed, the complications of the disease led to the decrease or loss of their income, thus increasing the IC (medical leave, time off, and productivity loss) of the patients treated with warfarin. In accordance with our findings, Deitelzweig et al. (2012), in a study in the United States, concluded that the use of NOACs such as rivaroxaban, apixaban, and dabigatran instead of warfarin reduced the annual medical costs of each patient by $179, $89, and $485, respectively [22].

In our study, the effectiveness outcomes were the no bleeding and prevention of ischemic stroke. The findings indicated that the treatment of AF patients with rivaroxaban and warfarin significantly decreased bleeding and stroke (81.9% vs. 44.4% for no bleeding and 98.6% vs. 95/7% for prevention of ischemic stroke). A study conducted by Hori et al. (2020) in Japan found that rivaroxaban reduced the risk of ischemic stroke compared to warfarin, and this study confirms the results of our research [9]. In a study by Kleintjens et al., the cost-effectiveness of rivaroxaban compared with warfarin was calculated for prevention of stroke in AF patients in the Belgian health system. According to the results, the internal bleeding in patients treated with rivaroxaban diminished from 0.063 to 0.048 compared to the patients treated with warfarin and caused an increase of 0.949 in QALY [13]. The results of a study conducted by Patel et al. (2011) in the United States indicated that rivaroxaban had almost the same effects as warfarin in the prevention of stroke and pulmonary embolism, and there was no significant difference in bleeding between the two groups. Meanwhile, severe bleeding was less frequent in the rivaroxaban group as compared to the warfarin group (0.5% intracranial bleeding was 0.5% vs. 0.7%, and severe bleeding was 0.2% vs. 0.5%) [24].

The results obtained in the present study indicated that the use of rivaroxaban in the treatment of AF patients was associated with fewer disabilities than the treatment with warfarin. Specifically, the patients undergoing the treatment with rivaroxaban were in a better condition in terms of moving around, self-care, daily activities, pain, and discomfort, as well as anxiety and depression compared to the patients treated with warfarin, which led to a decline in the mean score of QALYs in this group of patients. The mean values of the QALYs resulting from the health condition of each patient treated with rivaroxaban and warfarin were 0.5 and 0.33, respectively, with a significant difference being found between the utility scores (*P* < 0.05). In this regard, our results have been compared with the results of other studies. A study carried out by Craig et al. (2013) in the United States to compare the effectiveness and utility revealed that alteration of the treatment method and replacement of warfarin with rivaroxaban resulted in an increased QALY while bleeding decreased from 0.5 to 12.0. This is almost similar to the results of the study by Hori et al. (2020) and Salcedo et al. (2019) and Soyon et al. (2013) [9, 11, 21]. Also, Wang et al. (2014) calculated the cost-effectiveness of dabigatran and rivaroxaban in comparison with warfarin in patients with AF in Singapore. They concluded that the QALY values for warfarin, dabigatran with a dose of 110 mg, dabigatran with a dose of 150 mg, and rivaroxaban were 8.75, 8.73, 8.82, and 9.33, respectively. Their study indicated that rivaroxaban was a good alternative to warfarin in patients with AF [23]. The results of this study are the same as those conducted by Hori et al. (2020) and Salcedo et al. (2018), confirming the results of the present study.

The results of one-way sensitivity analysis showed that the ICER was not more sensitive to the majority of the parameters and confirmed the robustness of the study results. Further, the results of one-way sensitivity analysis for all three effectiveness outcomes revealed that ICER was the most sensitive to the cost of warfarin and the effectiveness of rivaroxaban;,while it showed little effect on other parameters. It suggests that rivaroxaban intervention is the dominant option and more cost-effective. Finally, the results of the studies by Soyon, Craig, Deitelzweig, and Kleintjens et al. in the United States and Belgium (2012 and 2013) [4, 13, 21, 22] indicated the cost-effectiveness of the treatment of AF patients with rivaroxaban, which is consistent with the results of the present study.

The strengths of this study are as follows: covering all the costs (DMC, DNMC and IC) and the use of cost and effectiveness data natively. However, one of the limitations of the present study is the short duration and nonuse of the Markov model. Another limitation was the generalizability of these findings, and we can generalize these results to other Iranian medical centers because of using warfarin and rivaroxaban to treat AF. But due to differences in quantities and prices, these results may not be comparable to other countries and should be used cautiously.

## 5. Conclusion

Considering the incremental cost-effectiveness ratio, rivaroxaban is more cost-effective than warfarin and can be a dominant alternative.

## Figures and Tables

**Table 1:** Direct and indirect costs of patients with atrial fibrillation treated with rivaroxaban and warfarin in 2019.

Cost type	Costs items	Rivaroxaban	Warfarin	*P* value
		Mean (PPP$)	Mean (PPP$)	
Direct medical costs	Physician visit	162	176	0.25
	Medication	1422	999	<0.001
	Diagnosis and lab services	33	217	<0.001
	Physiotherapy and other services^∗^	2425	2609	0.95
	Hospitalization	845	889	0.59
Direct nonmedical costs	Transportation, accommodation and meals	856	799	0.59
Indirect costs	Lost income	19531	20864	0.005
Total costs		25275	26554	0.004

^∗^Medical equipment, complementary medicine.

**Table 2:** Bleeding and ischemic stroke incidence of the AF patients treated with rivaroxaban and warfarin in 2019.

	Rivaroxaban		Warfarin	*P* value
Sex(male/female)	Male	%62.1	%46.9	0.11
	Female	%37.9	%53.1	
Mean age	66.7		65.1	0.72
CHADSVASC score	3.96		4.68	>0.05
Death	0		0	—
Mean of duration of medication	1	%67.2	%64.9	>0.05
	X > 1	%32.8	%35.1	
Place of living	Village	%52.8	%37.5	0.06
	City	%47.2	%62.5	
Illiterate	%52.4		%41.4	0.49
Ischemic stroke	%1.4		%4.3	>0.05
Hemorrhagic stroke	0		0	—
Myocardial infarction,	0		0	—
Intracranial hemorrhage	0		0	—
Life threatening nonintracranial hemorrhage	0		%2.8	0.31
Minor nonintracranial hemorrhage	Ecchymosis	%12.5	%36.1	<0.05
	Nosebleed	%2.8	%12.5	
	Blood in urine and stool	%2.8	%4.2	

**Table 3:** Results obtained from comparison of cost-effectiveness of two treatment methods for atrial fibrillation patients.

	Strategy	Cost (PPP$)	Effectiveness (based on clinical outcomes)		Incremental cost	Incremental effectiveness		ICER (incremental cost per extra success) PPP$
			Prevention of bleeding	Prevention of ischemic stroke		Prevention of bleeding	Prevention of ischemic stroke	
Cost-effectiveness analysis (CEA)	Rivaroxaban	25275	0.82	0.99	0	0	0	Dominant
	Warfarin	26554	0.44	0.96	1279	-0.38	-0.03	Dominated

**Table 4:** Results obtained from comparison of cost-utility of two treatment methods for atrial fibrillation patients.

	Strategy	Cost (PPP$)	QALYs	Incremental cost	Incremental QALYs	ICER (incremental cost per QALY gained) PPP$
Cost-utility analysis (CUA)	Rivaroxaban	25275	0.5	0	0	Dominant
	Warfarin	26554	0.33	1279	-0.17	Dominated

## Data Availability

The data for this study is not publicly available. The data that support the results of this study are available from the corresponding author, upon reasonable request.
